# Impact of the Perceived Mental Stress During the COVID-19 Pandemic on Medical Students' Loneliness Feelings and Future Career Choice: A Preliminary Survey Study

**DOI:** 10.3389/fpsyt.2021.666588

**Published:** 2021-06-08

**Authors:** Qiuyue Zheng, Xianhao Lin, Lin He, Thomas Freudenreich, Tao Liu

**Affiliations:** ^1^Department of Psychology, School of Health, Fujian Medical University, Fuzhou, China; ^2^Department of Marketing, School of Management, Zhejiang University, Hangzhou, China

**Keywords:** COVID-19, loneliness, career attitude, medical students, mental stress

## Abstract

The outbreak of the COVID-19 epidemic continues to unfold globally, and its negative impact on the public's mental health is starting to reveal. Serving as reserve talents for the healthcare system, medical students are not yet professionally matured enough to face one of the worst global public health crises. This may exert increased mental stress and loneliness feelings, which in turn negatively influence medical students' future career choice. To address the issue, we conducted three online survey studies investigating how the epidemic affects the mental health as well as career attitude of medical students in China during the COVID-19 pandemic outbreak. The results revealed preliminary evidence showing that the perceived stress induced by the COVID-19 epidemic might negatively affect medical students' future career choice, and the feeling of loneliness may play a mediating role. This study invites more attention to medical students' mental health during severe public health crisis such as the COVID-19 pandemic.

## Introduction

The outbreak of the COVID-19 epidemic has affected the lives of billions worldwide. In addition to causing economic upheaval and political unrest, the epidemic also poses a major challenge to public health—both physical and psychological (1, 2). Therefore, multidisciplinary researchers have been calling for attention to the mental health in various groups (3–5), among which understanding how the pandemic affects medical students is an important issue (6). The COVID-19 pandemic has brought a heavy burden on the global health system, and soon medical demand could outpace the medical capacity globally (7). This means we need all workforce available, and some countries have already moved their senior medical students to early graduation (8, 9). However, while being in training, medical students are missing the experience to deal with such severe situations, potentially leading to increased mental stress and in turn negatively influence their future career choice (10, 11).

In order to contain the coronavirus, governments across the world have enacted rigorous measures, such as the closure of borders, curfews, a general ban on assembly, and the closure of public places including parks, playgrounds, schools, universities, and shops. These control measures placed millions of people in isolation (12, 13), and may further result in an increase of mental health concern in loneliness (14–19). Loneliness is a painful emotional experience of a discrepancy between actual and desired social contact (20–23). During the COVID-19 pandemic, medical professions are encountering more challenges than ever (24, 25), and are over-identified accompanied by sensitivity to criticism, which may lead to increased loneliness feelings (26). Ample studies have demonstrated that loneliness may negatively impact individuals' future career orientation [e.g., (27, 28)]. Therefore, this study aimed to examine the issue of how the COVID-19 pandemic influences medical students' future career choice as well as the mediating role of loneliness.

## Perceived Mental Stress and Loneliness Feelings During the COVID-19 Pandemic

The outbreak of the COVID-19 pandemic has exerted severe pressure on global healthcare systems and stressed the already busy labor capacities within the systems, highlighting an urgent need for more medical and healthcare professions (7–9). Accordingly, countries around the world have reinstated retired doctors as well as clinical academics to clinical practice, and have given permission to final-year medical students to start working prior to their graduation (29). It is well-acknowledged that the COVID-19 pandemic exerted severe security challenges to the medical and healthcare professions, which increased their mental stress (25). As for the senior medical students, although not directly being at the frontlines of the pandemic, the medical students are still facing the risk of becoming infected with COVID-19 due to their rotation in the hospital which is accompanied by close contact with various kinds of patients. Therefore, the medical students may suffer increased mental stress during the COVID-19 pandemic.

Previous studies have demonstrated that the perceived mental stress may intensify feelings of loneliness (30–32). For instance, Yarcheski et al. (33) reported a significant positive correlation between perceived mental stress and loneliness. Similarly, in a recent review, Brown et al. (34) have also demonstrated positive associations between perceived mental stress and loneliness feelings. During the COVID-19 pandemic, medical and healthcare professions may have a sense of over-identification with the pandemic accompanied by sensitivity to criticism, which may increase the perceived mental stress as well as loneliness feelings (26).

## Medical Students' Career Orientation and Loneliness Feelings

Loneliness is a social construct, characterized by subjective feelings of social pain and/or isolation (32). The desire for social connections is a fundamental element in our survival gene (35, 36). Previous studies have examined the motivational aspects of individuals' interests in pursuing a career in medicine or healthcare to find new ways, not only to popularize these professions among adolescents and young adults but also to preemptively avoid future labor shortages in the healthcare sector during critical times such as the current COVID-19 pandemic. Literature has shown that intrinsic (i.e., personal interest, willingness to help), extrinsic (i.e., job security, financial remuneration), socio-demographic (i.e., gender, socio-economic status), as well as interpersonal factors (i.e., family, friends) could affect career preference of medical and healthcare students significantly (10, 11, 37, 38). The COVID-19 pandemic has exerted severe security challenges to the medical and healthcare professions, and may negatively influence their future career choice. For instance, the shortened internship experience may cause medical students to feel less prepared to enter the workforce. In addition, Isaac et al. (39) have revealed a negative relation between self-perception of social isolation, i.e., loneliness feelings, in medical students and their career intent.

## The Present Study and Hypothesis

This study aimed to examine the negative impact of perceived mental stress during the COVID-19 pandemic on medical students' attitude toward their future career choice, and to further uncover the mediating role of loneliness. A cross-sectional survey was conducted in March 2020 (Study 1), when the confirmed cases of the COVID-19 reached peak level in China, and a new survey (Study 2) with different measurements of perceived influence on medical students' career choice was collected in June 2020 to confirm the findings of Study 1. Study 3 was finally conducted in June 2020 with non-medical students as a control. Based on previous findings, we hypothesized that, compared with the non-medical students, the perceived mental stress by medical students during the COVID-19 pandemic would negatively influence their career choice, which was mediated by their feelings of loneliness. The respondents in three studies all gave their consent before filling in the questionnaire, and all studies were approved by the Institutional Review Boards of Fujian Medical University and School of Management, Zhejiang University.

## Study 1

### Methods

#### Sample and Procedure

We conducted a cross-sectional survey to 12 medical colleges and universities in China in March 2020, the worst period of the COVID-19 pandemic in China. We finally recruited 906 medical students (312 males, 594 females; 21.75 ± 1.90 years old; 3.35 ± 1.51 year of college).

### Measures

#### Demographic

Respondents were asked to provide their demographic and academic information such as gender, age, origin, major and college year.

#### Perceived Influence on Career Choice

Respondents were asked to directly rate the influence of the COVID-19 pandemic on their attitude toward future career choice as medical professions in a 5-point Likert scale (1 = “very positive influence,” 5 = “very negative influence”).

#### Perceived Stress

Respondents then scored their mental stress status using a 14-item Chinese Perceived Stress Scale (CPSS) (40) modified from Cohen et al. (41) with a 5-point scale (0 = “never” to 4 = “very often”). Sample items include “In the last month, how often have you been upset because of something that happened unexpectedly?.” In the present sample, the stress scale showed good internal consistency (Cronbach α = 0.87).

#### Feelings of Loneliness

Respondents also rated their loneliness feelings using a 20-item UCLA Loneliness Scale (UCLA-LS), version 3 (42). Sample items include “I can find companionship when I want it,” and “No one really knows me well.” Each item was rated on a 4-point scale, ranging from 1 = “never” to 4 = “most of the time.” The scale showed good internal consistency as well (Cronbach α = 0.92).

### Results

We firstly statistically assessed the common method bias using the Harman one-factor analysis. The first factor in our data explained only 26.27% of the variance, suggesting that common method bias was unlikely to confound the interpretations of our results.

The Pearson correlation analysis revealed significant positive relations between the scores on perceived mental stress (*M* = 38.66, *SD* = 6.55) and loneliness feelings (*M* = 40.66, *SD* = 9.10; *r* = 0.62, *p* < 0.001). In addition, the Pearson correlation analysis revealed significant positive correlations between the score of perceived influence on career choice (*M* = 2.14, *SD* = 1.06) and the respondents' age (*r* = 0.09, *p* = 0.007), college year (*r* = 0.17, *p* < 0.001), as well as their rating scores on perceived mental stress (*r* = 0.11, *p* = 0.001) and loneliness feelings (*r* = 0.13, *p* < 0.001). While controlling variables of age and college year, the partial correlation analysis still revealed significant positive correlations between the score of perceived influence on career choice and the mental stress score (*r* = 0.11, *p* = 0.001) as well as the loneliness score (*r* = 0.14, *p* < 0.001).

To further confirm the impact of perceived mental stress on respondents' career attitude and the mediating role of loneliness as well, we conducted a step-wise linear regression model taking age and college year as covariates. Table 1 shows detailed results of the regression analysis. Consistent with the correlation findings, perceived mental stress showed a positive relation with respondents' career attitude (beta = 0.017, *p* = 0.001) as shown in Model 2, which was non-significant (*p* = 0.426) while taking loneliness feelings (beta = 0.013, *p* = 0.005) into consideration, suggesting a potential mediating role of loneliness.

**Table 1:** Results of step-wise linear regression models predicting respondents' career attitude in study 1.

**Variables**	**Career preference**					
	**Model 1**		**Model 2**		**Model 3**	
	**Beta**	***p***	**Beta**	***p***	**Beta**	***p***
Constant	1.650	0.000	2.209	0.000	2.145	0.000
Age	−0.064	0.029	−0.060	0.040	−0.063	0.031
College year	0.182	0.000	0.177	0.000	0.183	0.000
Stress			0.017	0.001	0.005	0.426
Loneliness					0.013	0.005
*R^2^*	0.034	0.000	0.045	0.001	0.053	0.005
F	15.826	0.000	14.095	0.000	12.646	0.000
**Loneliness**	**Effect**		**SE**		**[LLCI, ULCI]**	
Total effect	0.017		0.005		[0.007, 0.027]	
Direct effect	0.005		0.007		[−0.007, 0.018]	
Indirect effect	0.012		0.004		[0.0034, 0.020]	

**Figure 1:**
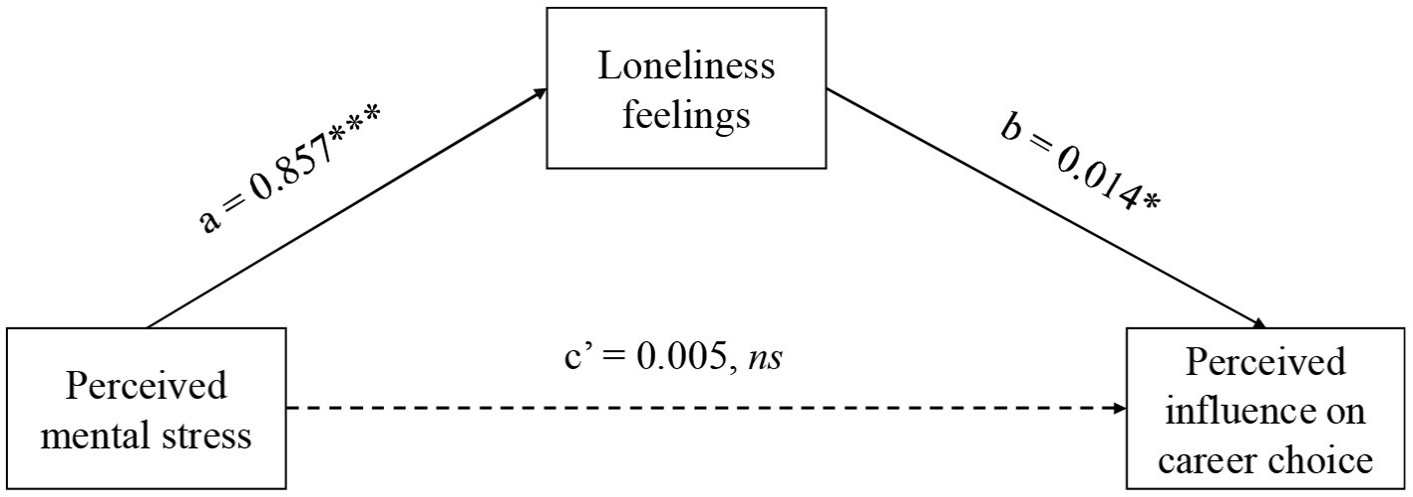
Results of mediation test using bootstrap method in Study 1. ns, *, *** represents *p* > 0.05, *p* < 0.05, *p* < 0.001, respectively.

The mediation test, using bootstrap (5000 times) by Model 4 in PROCESS for SPSS v3.5, confirmed that respondents' loneliness feelings fully mediated the relation between their perceived mental stress and career attitude (Figure 1). While taking loneliness feelings as the independent variable and perceived mental stress as the mediator, the same mediation test revealed significant direct effect [(LLCI, ULCI) = (0.002, 0.021)] but no significant indirect effect [(LLCI, ULCI) = (−0.003, 0.010)], confirming the unique mediating role of loneliness.

## Study 2

Study 1 showed preliminary evidence that the perceived mental stress by medical students during the COVID-19 pandemic negatively influenced their future career choice, which was mediated by loneliness feelings (although the effect size is relatively small). Study 2 was then designed to confirm the main findings revealed in Study 1 three months later in June 2020 with different measurements of perceived influence on future career choice. In addition, Study 2 could also address the issue of whether the negative stress-career relation was a short-term phenomenon or not, since the COVID-19 pandemic had been well-controlled in China in June.

### Method

We recruited 354 medical students from Fujian Medical University (196 males, 158 females; 21.09 ± 1.63 years; 2.86 ± 1.37 year of college). All respondents gave their consent and finished the same survey as used in Study 1, with different measurement of perceived influence of COVID-19 pandemic on their future career choice, and additional measurements of perceived risk during the COVID-19 pandemic.

In Study 1, we asked the respondents to directly rate the extent to which the COVID-19 pandemic influenced their future career attitude on a 5-point Likert scale, ranging from strong positive to strong negative influence. The results indicated that many of students rated positive influence, suggesting that the COVID-19 pandemic may exert impacts on medical students' future career attitude both negatively and positively. To focus on the negative influence, in Study 2 the respondents were asked to score two separate items that to what extent the COVID-19 pandemic negatively/positively influenced their future career attitude using a 3-point scale (1 = “no influence at all,” 3 = very negative/positively influence).

In addition, to examine whether the COVID-19 pandemic increased medical students' mental stress or not, we also asked the respondents to score the possibility that they could get infected by COVID-19 using a 4-point scale (1 = “almost impossible,” 4 = “highly possible”).

### Results

The Harman's single-factor analysis showed that the first factor in our data explained only 27.03% of the variance, suggesting that common method bias was unlikely to confound the interpretations of our results.

As expected, the self-reported possibility of infection (*M* = 2.06, *SD* = 0.77) positively correlated with respondents perceived mental stress (Cronbach α = 0.857; *M* = 38.18, *SD* = 7.21; *r* = 0.12, *p* = 0.023), indicating that the COVID-19 pandemic indeed increased medical students' mental stress. Consistent with the findings revealed in Study 1, the Pearson correlation analysis also revealed a significantly positive correlation between the scores on perceived mental stress and loneliness feelings (Cronbach α = 0.818; *M* = 41.52, *SD* = 10.19; *r* = 0.633, *p* < 0.001). Focusing on the perceived negative influenced of the COVID-19 pandemic on medical students' future career choice (*M* = 1.48, *SD* = 0.53), the Pearson correlation analysis revealed significant positive correlations between the negative career influence and their age (*r* = 0.12, *p* = 0.021) and college year (*r* = 0.20, *p* < 0.001). More importantly, the negative career influence also positively correlated with the perceived mental stress (*r* = 0.12, *p* = 0.025) as well as the loneliness feelings (*r* = 0.20, *p* < 0.001). The partial correlation analysis, taking age and college year as control variables, revealed similar results (stress-career: *r* = 0.11, *p* = 0.043; loneliness-career: *r* = 0.18, *p* = 0.001).

**Figure 2:**
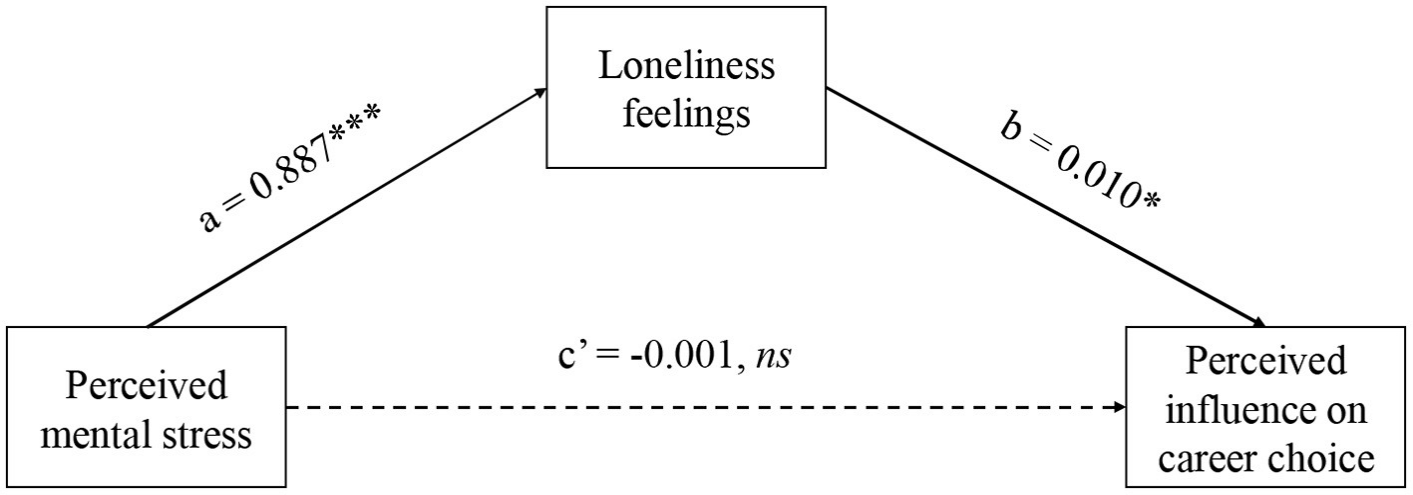
Results of mediation test using bootstrap method in Study 2. ns, *, *** represents *p* > 0.05, *p* < 0.05, *p* < 0.001, respectively.

The step-wise linear regression models also revealed significant positive relation between the perceived mental stress and the negative career influence (beta = 0.008, *p* =.040), which was non-significant (*p* = 0.879) while taking loneliness feelings (beta = 0.010, *p* =.006) into consideration. Consistently, as Table 2 shows, the mediation test using bootstrap method (5,000 times, in PROCESS for SPSS v3.5) further revealed that the respondents' loneliness feelings fully mediated the relation between perceived mental stress and negative career influence (Figure 2).

**Table 2:** Results of linear regression models predicting respondents' career attitude using perceived stress in study 2.

**Variables**	**Career preference**					
	**Model 1**		**Model 2**		**Model 3**	
	**Beta**	***p***	**Beta**	***p***	**Beta**	***p***
Constant	1.914	0.000	1.552	0.007	1.467	0.010
Age	−0.036	0.227	−0.032	0.282	−0.030	0.304
College year	0.109	0.002	0.103	0.003	0.095	0.006
Stress			0.008	0.043	−0.001	0.879
Loneliness					0.010	0.006
*R^2^*	0.042	0.001	0.053	0.043	0.073	0.006
F	7.708	0.000	6.555	0.000	6.909	0.000
**Loneliness**	**Effect**		**SE**		**[LLCI, ULCI]**	
Total effect	0.008		0.004		[0.001,0.015]	
Direct effect	−0.001		0.005		[−0.010, 0.009]	
Indirect effect	0.009		0.003		[0.003, 0.015]	

## Study 3

Study 3 examined influence of the COVID-19 pandemic on non-medical students' future career choice as a control condition. We recruited 175 non-medical students (78 males, 97 females; 23.14 ± 2.44 years; 5.50 ± 2.36 year of college due to involvement of graduated students) during the same period as the Study 2 (in June 2020) in Zhejiang University. All respondents gave their consent and finished the same survey as used in Study 1.

The Harman's single-factor analysis showed that the first factor in our data explained only 32.27% of the variance, suggesting that common method bias was unlikely to confound interpretations of our results. Consistent with the findings of Study 1 and Study 2, a positive correlation was revealed between the scores of mental stress (Cronbach α = 0.627; *M* = 40.65, *SD* = 8.02) and loneliness feelings (Cronbach α = 0.671; *M* = 52.95, *SD* = 5.80; *r* = 0.28, *p* < 0.001). Even though, the perceived influence of the COVID-19 pandemic on non-medical students' future career choice (*M* = 3.58, *SD* = 1.12) showed no significant correlations with either mental stress (*r* = −0.06, *p* = 0.466) or loneliness feelings (*r* = 0.034, *p* = 0.658).

## General Discussion

Understanding the issue of how the COIVD-19 pandemic influences medical students' mental health and future career choice as medical and healthcare professions is an important topic in the current situation. Through 3 studies, we revealed that the perceived mental stress induced by the COVID-19 pandemic may negatively influence medical students' future career choice, which is mediated by their feelings of loneliness. Ample studies have examined the issues of how the COVID-19 pandemic affects individuals' mental health [e.g., (3–5)], however, very few studies focused on medical students' career attitude as well as the role of loneliness. Previous studies have identified that loneliness significantly impacts individuals' career orientation (27, 28, 39, 43). This research contributes to the literature understanding the impacts of the COVID-19 pandemic on medical students' mental health and career orientation, and calls for more attention to medical students' mental health and future career orientation during severe public health crisis such as the COVID-19 pandemic, emphasizing the importance of loneliness. Practically, both medical universities and governments should provide more psychological supports to medical students reducing their mental stress as well as the loneliness feelings in particular.

We mainly conducted two cross-sectional survey studies in March and June 2020 in China. March 2020 was the first month when the Chinese government activated level-1 public health emergency responses in 31 provincial-level regions in mainland China (44). June 2020 was the first month when people returned to the post-pandemic “normal” life in China after well-controlling the COVID-19. The two studies consistently revealed that the COVID-19 pandemic negatively impacts medical students' mental stress as well as feelings of loneliness. This prevalence psychological response in the time of COVID-19 pandemic is consistent with the general public (13). For instance, Romeo et al. (2) have identified several factors that could predispose University students to a high risk of developing mental health symptoms as a consequence of the COVID-19 pandemic.

More importantly, we identify a critical role of loneliness suffered by medical students in their future career choice during the public health crisis, although the effect size is relatively small. One possible explanation is that the Chinese government adopted rigorous control measures timely and provided huge supports to medical and healthcare systems, reducing the negative influences of the COVID-19 pandemic. In the survey of Study 2, medical students reported great confidence in the Chinese government (3.81 ± 0.47), while rating the item of “how much confidence do you have in the government policy supporting medical and healthcare systems” on a 4-point scale (1, not at all; 4, very much). Even though, our studies with 1260 medical students consistently showed that the COVID-19 pandemic increased medical students' mental stress, which in turn aggravated their feelings of loneliness and may further negatively impact their future career choice. Future studies are needed to confirm the negative career influence from the severe public health crises.

Previous studies focusing on medical students' career choice have mainly emphasized intrinsic and extrinsic factors such as early specialty interest, self-competency, clinical exposure, and patient-doctor relationship (45), but neglected psychological factors such as loneliness. This research appeals to pay more attention to medical students' mental health during a serious public health crisis, emphasizing their loneliness feelings in particular, and its negative impact on their career choice as medical and healthcare professions.

It is noteworthy that Study 3 with non-medical students did not show the same stress-loneliness-career relation as revealed in medical students. Although the sample size of Study 3 was relatively small, even a clear tendency was not found. One possible explanation is that the COVID-19 pandemic not only poses a major challenge to public health, but also causes massive economic and political unrest, which further negatively impacted non-medical students' work situation. In order to contain the virus, governments across the world have enacted measures such as the closure of borders, curfews, a general ban on assembly, placing millions of people in isolation and subsequently damaging their own commercial system in the process. In this case, non-medical students may perceive feelings of uncertainty when it comes to job hunting. Thus, the perceived mental stress and feelings of loneliness may contribute little to their career choice. Future study is needed to address the issue of how the COVID-19 pandemic influences non-medical students' mental health and career situation.

The present research has several limitations as well. First, this research was a cross-sectional design, and hence cannot conclude a causal relationship between variables. Second, the samples were mainly recruited from the East coast of China. More studies should be done to examine the influence of the COVID-19 epidemic on medical students' mental health and career choice globally. More importantly, researchers should pay more attention to other critical factors such as poverty and lack of resources and examine how such factors influence the stress-loneliness-career relation in medical students. Third, the measurement of career influence had only one item, the robustness of the results should be confirmed in future studies. Even though, the respondents were asked to directly rate the scores of how the COVID-19 epidemic impacts their future career choice as medical and healthcare professions, providing preliminary evidence emphasizing the importance of the potential negative influence of the COVID-19 epidemic on medical students' mental health and career choice. Fourth, this research did not collect comparable samples from both medical and non-medical students in one study, limiting our conclusion on the uniqueness of the stress-loneliness-career relation induced by the COVID-19 epidemic in medical students, which should be confirmed in the future study.

## Data Availability Statement

The raw data supporting the conclusions of this article will be made available by the authors, without undue reservation.

## Ethics Statement

The present studies were approved by the Ethical Review Boards of Fujian Medical University and School of Management, Zhejiang University. The patients/participants provided their written informed consent to participate in this study.

## Author Contributions

TL designed the theoretical framework and analyzed the data. QZ, XL, and LH collected the data. All authors wrote the manuscript.

## Conflict of Interest

The authors declare that the research was conducted in the absence of any commercial or financial relationships that could be construed as a potential conflict of interest.

## Abbreviations

COVID-19: The 2019 Novel coronavirus diseases.
